# Assessing the feasibility of use and content validity of ICECAP-CPM with bereaved family members of young people who died from serious illness: a UK think-aloud study

**DOI:** 10.1186/s12904-026-02118-9

**Published:** 2026-04-28

**Authors:** Isabella Floredin, Paul Mark Mitchell, Samantha Husbands, Susan Neilson, Joanna Coast

**Affiliations:** 1 Health Economics and Health Policy (HEHP@Bristol), Population Health Sciences, Bristol Medical School, University of Bristol, 1-5 Whiteladies Road, Bristol, BS8 1NU UK; 2 Institute of Clinical Sciences, University of Birmingham, Edgbaston, Birmingham, B15 2TT UK

**Keywords:** End-of-life care, Hospice care, Palliative care, Adolescents, Young people, Family members, Bereavement, Outcome assessment

## Abstract

**Background:**

The ICECAP-Close Person Measure (CPM) is a self-complete measure developed for use in economic evaluation to capture capability outcomes for those close to individuals at the end of life. Its appropriateness for use with close persons of young people, aged 14–25, at the end of life has not yet been assessed. An initial assessment of its use with bereaved family members of young people with serious illness was undertaken.

**Methods:**

To assess feasibility of use and content validity of the measure, bereaved participants were asked to ‘think aloud’ when completing the ICECAP-CPM. Interview transcripts were examined for errors in comprehension, retrieval, judgement and response by four raters. Qualitative data explored reasons for errors during measure completion and participants’ views about the measure.

**Results:**

Nine cognitive interviews were undertaken with parents (five) and siblings (four) of young people who had died. The measure appeared to be understood by the majority of participants, but there were errors in completion (18%) and struggles (4%). Participants suggested the measure was relevant to their experience and covered broad areas important to them.

**Conclusions:**

Although completion errors were identified, the measure appears relevant for use with bereaved family members of young people with serious illness.

**Supplementary Information:**

The online version contains supplementary material available at 10.1186/s12904-026-02118-9.

## Background

End-of-life care (EoLC) and bereavement have a significant impact on those close to young people (YP) approaching death [1–3]. Provision of support to family members or close friends (collectively referred to as “close persons”) can be beneficial across the illness trajectory, from the point of diagnosis to the time of death and in bereavement [4]. Support for close persons might include respite care, psychological and bereavement support and advice on finances [5]. However, many of these needs are often left unmet partly due to the focus being on caring for the affected YP [6–8].

Health economic evaluations are widely used in a number of countries, such as the UK [9], Canada [10], Australia [11] and Thailand [12], to compare the costs and outcomes of alternative interventions and guide effective resource allocation [13]. Evaluations often overlook the impact of interventions on persons beyond the individual patient [14]. Some evaluations consider carers but do not extend to other close persons [15–17]. Moreover, key aspects of EoLC that extend beyond health functioning that may be important to close persons, such as communication with services [18], are often not captured in health economic evaluations. Not including such effects for the purpose of allocating resources may result in an under-provision of care if interventions are perceived as not cost-effective [19, 20].

The capability approach offers an alternative framework for economic evaluation of health and care interventions capturing broader wellbeing [21, 22]. The approach focuses on what people are able to be and do in life which may be particularly relevant for evaluating EoLC interventions and interventions for close persons [23–26].

The ICECAP – Close Person Measure (CPM) is a self-complete economic measure developed to capture capability outcomes for those close to individuals at the end of life (EoL) [24]. Its six dimensions were developed through qualitative work with close persons of an older adult patient population (age range of decedent 40 to 80+) and cover communication, privacy and space, practical support, emotional support, preparing and coping, and emotional distress. Its properties have not yet been assessed for close persons of YP, aged 14–25, at the EoL. Close persons of YP may have different experiences and needs to close persons of older adults at the EoL.

An important criterion for the initial psychometric assessment of a measure is feasibility, that is the extent to which the measure can be used in practice in the relevant context [27]. Another key measurement property to test is content validity, particularly when the measure is intended to be used within a different context. Content validity concerns the extent to which the content of the measure is relevant and important to the specific intervention or population [28, 29].

This paper reports a UK-based preliminary investigation of the feasibility and content validity of ICECAP-CPM for bereaved close persons of YP, aged 14–25 years, at the EoL.

## Methods

Qualitative methods were used to explore feasibility of use and content validity of ICECAP-CPM in bereaved close persons of YP with serious illness. Consistent with previous studies undertaken to assess ICECAP measures [30–32], cognitive interviewing – using the think-aloud technique [33] followed by semi-structured interviewing – was employed to assess the appropriateness of the measure in this context.

Interviews were conducted with bereaved parents and siblings of YP aged 14–25 who had died from serious illness. Recruitment and data collection occurred during the COVID-19 pandemic which had a significant impact on recruitment and data collection. Initial recruitment plans focused on recruitment through hospices and charitable organisations. These services were affected by the pandemic and health providers were under extreme workload pressures. As a result, recruitment strategies had to be adapted to include recruitment through social media and use of online interviews. Participants were recruited from across the UK through hospices, charitable organisations offering bereavement support and social media with a maximum variation approach to sampling to capture different perspectives from a range of backgrounds, conditions and care settings. Given the limited pool of potential participants, all eligible respondents were included. Further participants were recruited using snowball sampling.

Individuals within 6 months of bereavement were not recruited to minimise the potential impact of early grief [24]. A protocol was developed for the interviewer to address potential participant distress [34]. Participants gave informed consent to participate in the study before taking part.

Interviews were conducted online using Microsoft teams and audio recorded. Interviews consisted of two parts. Part one involved in-depth interviews to develop conceptual attributes for a new EoLC measure for YP. Findings from part one are reported in a previous study [25]. Even though the interviews were conducted remotely, part one enabled the researcher to develop initial rapport with participants and ease them into part two which involved asking specific questions around EoLC. Part two focused on participants’ own experiences to assess the appropriateness of ICECAP-CPM. In part two, participants first completed ICECAP-CPM [24], consisting of six attributes with five response levels, while thinking aloud to verbalise their thoughts. During the think aloud part of the interview the researcher remained silent unless the participant was silent for longer than ten seconds at which point the researcher prompted the participant to keep thinking aloud. Participants were then asked about their views of the measure, the extent to which they felt each of the measure dimensions were comprehensible and relevant to their experience as a close person and to clarify any issues they had with measure completion, such as in relation to the measure instructions or ticking between two of the available response options (topic guide in Supplementary file 1). Following data collection, the researcher went through a debriefing statement with participants that included information about free national support services the participant may have wished to access.

Interviews were transcribed verbatim and imported into NVivo12 for analysis. Transcripts were rated by four independent reviewers [initials of reviewers removed for peer-review] to identify items where participants experienced difficulties (error or struggle) when completing each question. Tourangeau et al.’s model for survey responses [35] was used as a rating framework for errors. Errors were based on four criteria: question comprehension in the way the measure intended (comprehension); retrieval of appropriate information to answer the question (retrieval); correct judgement on how to use recalled information to answer the question (judgement); use of information in a way that gives a valid response to the question (response) (see Supplementary file 2). A ‘struggle’ category was also included, following earlier studies [36], to identify areas where there was no error and the participants reached an appropriate response but had difficulty answering the question.

Each rater used the relevant section of the transcript and information about the participant’s questionnaire response to assess whether one of the four types of errors was present or if there was a ‘struggle’ (Scoring Sheet in Supplementary file 2). Raters discussed areas of discrepancy in their independent responses to reach a consensus on the final number and type of errors or struggles.

Constant comparison was used to analyse data from the semi-structured interviews [37] to identify and compare recurring themes across participants on each item’s relevance to participants’ experiences.

## Results

Nine interviews, five with parents and four with siblings of YP who had died were undertaken (Participant characteristics in Table 1), between November 2021 to September 2022.

**Table 1:** Participant characteristics

Participant characteristics (*n* = 9)		
Age group (years)		
20–29	4	
40–49	1	
50–59	4	
Gender		
Male		2
Female		7
Relationship to young person		
Mother		5
Brother		2
Sister		2
Months since bereavement		
6–11		2
12–18		5
> 18		2
Recruitment method		
Charities		3
Social media		4
Snowball		2
Ethnicity		
white British		6
black		3
Deprivation level (IMD)		
1–3 (most deprived)		6
4–7		1
8–10 (least deprived)		2
Age group (years) of associated young person at death		
14–17		2
18–25		7
Gender of associated young person		
Male		3
Female		6
Health condition of associated young person		
Congenital		1
Cancer		5
Circulatory		1
Neurological		2

## Measure completion rating

All respondents completed six ICECAP-CPM attributes. Forty-two of 54 of responses (78%) were free of error or struggle. Errors were identified for 10 (18%) and struggles in 2 (4%) responses following rater consensus. At least one type of error or struggle was identified for six respondents (67%). Eight out of the 10 errors were response errors - five were identified in one respondent who gave in-between response level answers to five out of six attributes (between the bottom two response levels across all five attributes).

### CP06

*“…most of the time I would say it was between D and E…”*.

No judgement errors were identified. At least one type of error was identified in all attributes except for ‘Preparing and Coping’. The ‘Communication’ attribute had most errors and struggles (Table 2).

**Table 2:** Errors and struggles identified by type across ICECAP-CPM attributes

Error type	ICECAP- CPM attributes						
	Communication	Privacy and Space	Practical support	Emotional support	Preparing and coping	Emotional distress	Total
Comprehension	0	0	1	0	0	0	1
Retrieval	0	0	1	0	0	0	1
Judgement	0	0	0	0	0	0	0
Response	3	1	1	2	0	1	8
Struggle	1	0	0	0	0	1	2
Total	4	1	3	2	0	2	12

All response errors were due to respondents wanting more flexibility to provide different responses for different services received at different times. For instance, in the ‘Communication’ attribute, it was difficult for respondents to ‘aggregate’ their experiences over time into a single response. Communication at different time points and with different services varied. Respondents suggested specifying the time period and services in the question and if measuring post-death experiences, adjusting the question to highlight the EoL period.

### CP18

*“…it might be good for them to have an option […] like communication with providing care services perhaps as an option for different hospitals ….”*

### CP17

*“…he had such a long journey […] some of the times it was fine. Some of the times there was no communication […] I just wouldn’t be able to pinpoint which one of those I would really be able to say…”*.

### CP07

*“…I think it has varied about who you would be speaking to…”*.

In the ‘Practical support’ attribute, the comprehension error identified was due to the respondent misinterpreting the question, focusing on hospital-provided information around support rather than actual support available or experienced, possibly influenced by limited involvement of support services.

### CP13

*“… they gave us some information […] [they] handed you the practical support*,* and then you had to go away and read it…”*.

The retrieval error identified in this attribute was because the respondent, who was a sibling, lacked details around support, as their mum was the primary caregiver.

### CP15

*“…I wouldn’t really know because it was my mum that was basically doing all the stuff …”*.

For the ‘emotional distress’ attribute the struggle was due to the respondent’s experience with the sudden death of the YP.

### CP18

*“…because it was sudden […] when I first looked at [the emotional distress question] I wasn’t really sure how I could relate to that …”.*

## Views of respondents

Overall, most respondents (*n* = 7) viewed the measure positively and as relevant to their experience as a close person.

### CP14

*“…all these […] are questions that are really important …”*.

### CP07

*“…it has covered the practical and the emotional side…”*.

### CP13

*“…it was questions that needed to be asked […] it is all relevant…”*.

Some (*n* = 5) found completing the measure helpful as it allowed them to share their experiences and have their feelings acknowledged.

### CP06

*“… It is good to be able to […] say what we have been feeling […] because we haven’t had the opportunity to say all that…”*.

### CP17

*“…it’s helped validate that it is an awful thing to have happen to you*,* when your child dies. And that people do care …”*.

Others (*n* = 2), however, found completing the measure difficult due to its sensitive topic and having to recall traumatic events to be able to respond.

### CP15

*“…I found it quite difficult […] it brought back memories…”*.

## Discussion

ICECAP-CPM attributes appeared to be understood by the majority of participants. Participants felt the measure covered broad areas important to them, addressing both practical and emotional aspects of their experiences.

Positive feedback from participants on their feelings around being acknowledged mirrors carers’ reports in social care surveys, where participants were surprised by the focus on their own wellbeing rather than the patient’s, which was their previous experience when completing healthcare questionnaires [38, 39]. ICECAP-CPM respondents’ views on being heard and acknowledged, further support the value of capturing outcomes beyond the patient in evaluations.

Some participants struggled to complete the measure due to the sensitive nature of the topic, a challenge noted in previous studies using similar measures [26, 30, 31]. Recommendations suggest using a distress protocol to support respondents completing EoLC measures if needed [31, 34]. This study reiterates the use of a protocol for ICECAP-CPM completion, especially for close persons of YP, given the intensity of grief experienced in this group [2, 40].

The completion error rate (18%) was high compared to similar think-aloud work with hospice care patients (3.9%) [30] and patients experiencing end-stage organ failure (5.7%) [31]. While there are no recommendations on adequate sample size for cognitive interviews, and studies on sensitive topics often involve low numbers of participants [34, 41–43], the higher error rate may be due to the small sample with a high number of errors identified from one respondent.

The high error rate, particularly response errors, may also be influenced by the post-hoc nature of measure completion in this study (following YP’s death) and differences in EoLC timing, with adults often receiving care later in illness and for shorter periods than YP [18]. Additional instructions highlighting the timing of the experience the measure is looking at (for example adding, ‘Thinking about the last few days of the young person’s life…’) may assist with the error rate.

Completion of the measure following the death of the YP may help to explain why the ‘Preparing and Coping’ attribute was error-free, as all respondents had already managed the YP’s post-bereavement practical arrangements and were grieving. This contrasts with response type errors in a study [31] with end-stage organ failure patients, where respondents had not yet considered the ‘Preparation’ attribute.

Further work to assess the appropriateness of the measure in this context is needed, with larger samples generally, and particularly with fathers and friends as there were no participants from these two groups and their relationship and role may differ to those of mothers and siblings. The higher proportion of female participants is not surprising and has been evident in many studies in EoLC where mothers constitute the majority of the sample [44]. Future work could also consider adapting the measure to be clearer about the timescales that are being considered, particularly for bereaved individuals rather than those completing the measure during the end-of-life period.

The voices of those who are ‘experts by experience’ [45] in terms of caring for and about a YP using EoLC have been included in this study to better understand their experiences. These voices contribute further empirical evidence that can help influence how services are designed to better meet the needs of those close to YP at the EoL.

## Conclusion

This preliminary assessment of ICECAP-CPM suggests that the measure appears to capture important aspects of the experience of bereaved close persons of YP but the timescales being considered in the measure may need to be adapted for this group.

## Supplementary Information

Supplementary Material 1.

Supplementary Material 2.

## Data Availability

The data generated and analysed during the current study are not publicly available due to them containing information that could compromise research participant privacy but are available from the corresponding author [IF] on reasonable request.

## Abbreviations

EoLC: End-of life-care
YP: Young people
CPM: Close Person Measure
EoL: End of life
