# Supplementary material for: Assessing the feasibility of use and content validity of ICECAP-CPM with bereaved family members of young people who died from serious illness: a UK think-aloud study
Source: BMC Palliat Care. 2026 Apr 28;25:168. doi: 10.1186/s12904-026-02118-9 (PMC13255345; doi:10.1186/s12904-026-02118-9)
Supplement: Supplementary file 1 — Supplementary Material 1. [file 12904_2026_2118_MOESM1_ESM.docx]

Supplementary File 1: Interview topic guide

**‘THINK ALOUD’ TECHNIQUE AND SEMI-STRUCTURED INTERVIEW**

- Provide the participant with the ICECAP-CPM and explain that the questionnaire asks about the quality of life for bereaved close persons, and it has been developed for use in older adult population, so we are interested to know if it can be used for those who have been close to a younger person who has died.
- Ask the participant to verbalise their thoughts by thinking out loud whilst completing the measure. Also ask the participant to say which choice they would pick for each question on the questionnaire whilst thinking out loud. Explain that we are interested in what they are thinking while they are responding to the questions
- Remain silent unless the participant is silent for longer than 10 seconds at which point ask the participant to keep thinking aloud
- Following completion of ICECAP-CPM ask the participant:
  - **How did you find completing the questionnaire?** Researcher will explore thoughts the participant expressed whilst completing the questionnaire
  - **Have you found any aspects of the questionnaire challenging? If so, please explain.** Researcher will explore and clarify any issues that came up during the ‘think aloud’ task.
  - **How relevant was each question to your experience as a close person?** Is there a question that you would add to the questionnaire? What would that be? / Is there any question you think is not relevant to you? Why?
