# Supplementary material for: Assessing the feasibility of use and content validity of ICECAP-CPM with bereaved family members of young people who died from serious illness: a UK think-aloud study
Source: BMC Palliat Care. 2026 Apr 28;25:168. doi: 10.1186/s12904-026-02118-9 (PMC13255345; doi:10.1186/s12904-026-02118-9)
Supplement: Supplementary file 2 — Supplementary Material 2. [file 12904_2026_2118_MOESM2_ESM.docx]

# Supplementary File 2: Rating framework for errors

# Think Aloud Scoring Sheet ICECAP-CPM

| *ID* |  |  |  | ICECAP-CPM |  |  |  |  |  |  | **NB: 5=best 1=worst** |  |  |
| --- | --- | --- | --- | --- | --- | --- | --- | --- | --- | --- | --- | --- | --- |

| Rater | ^1^ | ^2^ | ^3^ | ^4^ |
| --- | --- | --- | --- | --- |

| No | **Item** | ***(a) Comp*** | (b) Retrieval | ***(c) Judge*** | ***(d) Response*** | **(e) Struggle** | **(f)** Comments |
| --- | --- | --- | --- | --- | --- | --- | --- |
| 1 | **Good communication** with those providing care services |  |  |  |  |  |  |
| 2 | **Privacy and Space** (to be with the loved one) |  |  |  |  |  |  |
| 3 | **Practical support** |  |  |  |  |  |  |
| 4 | **Emotional support** |  |  |  |  |  |  |
| 5 | Being able to **prepare and cope** |  |  |  |  |  |  |
| 6 | **Being free from emotional distress** related to the condition of the decedent |  |  |  |  |  |  |

1. *Comprehension*
   - - Misunderstanding of a word, phrase, or response option
     - Words or phrase that the respondent does not understand
2. *Retrieval*

- Recall error or a miscalculation of the time frame stated

1. *Judgement*
   - Response does not match that of the researcher’s intent for the question
   - Recalled relevant experiences that the respondent questions as irrelevant or inadequate
2. *Response*
   - Respondent’s desired response is missing from the written response options
   - Response which is felt to be a socially desirable answer
   - Response is inconsistent with the personal experience expressed
   - Respondent’s answer is inconsistent with previous answers
3. Struggle

- The respondent has had difficulty answering the question, even when final response is correct
